# New Steroidal Selenides as Proapoptotic Factors

**DOI:** 10.3390/molecules28227528

**Published:** 2023-11-10

**Authors:** Izabella Jastrzebska, Natalia Wawrusiewicz-Kurylonek, Paweł A. Grześ, Artur Ratkiewicz, Ewa Grabowska, Magdalena Czerniecka, Urszula Czyżewska, Adam Tylicki

**Affiliations:** 1 Faculty of Chemistry, University of Białystok, Ciołkowskiego 1K, 15-245 Białystok, Poland; p.grzes@uwb.edu.pl (P.A.G.); artrat@uwb.edu.pl (A.R.); 2 Department of Clinical Genetics, Medical University of Białystok, Waszyngtona 13, 15-089 Białystok, Poland; natalia.wawrusiewicz-kurylonek@umb.edu.pl; 3 Doctoral School of Exact and Natural Sciences, University of Bialystok, K. Ciolkowskiego 1K, 15-245 Bialystok, Poland; e.grabowska@uwb.edu.pl; 4 Faculty of Biology, University of Białystok, Ciołkowskiego 1J, 15-245 Białystok, Poland; m.siemieniuk@uwb.edu.pl (M.C.); urszula.czyzewska@uwb.edu.pl (U.C.)

**Keywords:** antimetabolites, cell growth inhibition, gene expression, HeLa cells, in vitro culture

## Abstract

Cytostatic and pro-apoptotic effects of selenium steroid derivatives against HeLa cells were determined. The highest cytostatic activity was shown by derivative **4** (GI_50_ 25.0 µM, almost complete growth inhibition after three days of culture, and over 97% of apoptotic and dead cells at 200 µM). The results of our study (cell number measurements, apoptosis profile, relative expression of apoptosis-related *APAF1*, *BID*, and mevalonate pathway-involved *HMGCR*, *SQLE*, *CYP51A1*, and *PDHB* genes, and computational chemistry data) support the hypothesis that tested selenosteroids induce the extrinsic pathway of apoptosis by affecting the cell membrane as cholesterol antimetabolites. An additional mechanism of action is possible through a direct action of derivative **4** to inhibit *PDHB* expression in a way similar to steroid hormones.

## 1. Introduction

Selenosteroids (SeSt) are compounds formed by attaching a selenium-moiety to a steroid molecule [1]. Taking into consideration their structure, we can identify two groups of seleno-compounds: first with selenium directly connected to the steroid molecule to form a selenide and second where selenium is added to the steroid in the form of an organic (for example selenourea, benzsoselenazolones) [2,3] or inorganic (for example selenocyanate) [4] moiety. During the recent decades, much information about methods of SeSt synthesis has appeared [1]. Recently, selenocyano groups were directly introduced into pregnenolone, estradiol, and estrone [5] as well as using cholesterol and norcholesterol [6]. The results of that research indicate that some of the obtained SeSt exhibit antiproliferative properties against cancer cells. So far, little research has focused on the effects of SeSt on cells, and most studies have been limited to determining the effect of these compounds on the rate of cell proliferation with the determination of GI50 (growth inhibition—concentration of the compound resulting in a 50% reduction in the number of cells) or IC50 (inhibition concentration—concentration of compound causing 50% of metabolism inhibition) values in various in vitro tumor models [3,4,7,8] and bacteria [9]. Recent publications have also documented the inhibitory effects of selenosteroids on human tumors in animal models [5,6,10]. There is also information about the antioxidant actions of some SeSt [2,3]. Despite this prior knowledge about the effects of these compounds on cell cultures, there is still insufficient information to explain mechanism of their action at the cell biology level.

Last year a new method of selenosteroids synthesis was described [8]. This method employs a one-step, eco-friendly synthesis that bypasses some steps connected with the bad smell and the reactivity of commonly used seleno-reagents. Furthermore, the method demonstrated the potential to prepare libraries of steroids variously and selectively decorated with different organochalcogen moieties. Using this method, several seleno-steroidal derivatives were obtained based on the same steroid core containing selenium connected to the A ring of a steroid [11].

**Scheme 1:**
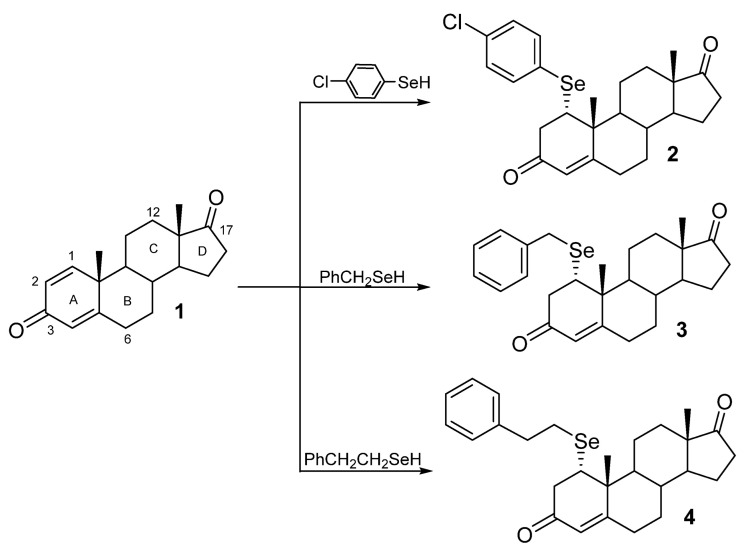
Synthesis of selenosteroids **2**–**4**.

Our study aimed to compare the effects of obtained SeSt (Scheme 1) on HeLa cancer cell line in vitro in relation to other SeSt known from the literature and propose the mechanism of their action. In addition, having the above-mentioned model of SeSt structures, we were able to check their impact on cancer cells concerning chemical structure (the presence of a chlorine atom and the length of the linker between phenylene group and steroid core). Answers to these questions may guide further syntheses to optimize anti-proliferative effects. Knowing the previous data on the antiproliferative effect of SeSt on cells [1,4,5,6,10], we assumed that the tested compounds may have pro-apoptotic properties. From the point of view of their medical use, it is important which apoptotic pathway is activated [12]. In relation to the above, we consider the assumption that SeSt can act as cholesterol antimetabolites, so by interacting with the cell membrane, they can promote the extrinsic apoptosis pathway and can interact with the mevalonate pathway of steroid synthesis [13].

To test the above hypotheses, in addition to research on the HeLa cell growth rate under the influence of SeSt and their apoptosis profile, we also examined the expression of selected genes related to cholesterol synthesis and cell apoptosis as well as the possibility of SeSt interaction with the cell membrane using computational chemistry models.

To investigate the cholesterol synthesis pathway, we chose four genes: two enzymes that are key rat-limiting factors of sterol synthesis in human cells [11], *HMGCR* encoding 3-hydroxy-3-methylglutaryl-CoA reductase, which produces mevalonate, and *SQLE* encoding squalene monooxygenase, which catalyzes the oxidation of squalene to 2,3-epoxysqualene; the other two enzymes are *CYP51A1* encoding monooxygenase from the cytochrome P450 superfamily, which participates in cholesterol synthesis by removal of the methyl group at C-14 position from lanosterol [14], and *PDHB* encoding E1 beta subunit of pyruvate dehydrogenase complex which generates acetyl-CoA [15], a key substrate for cholesterol synthesis, by pyruvate oxidative decarboxylation.

Among the genes responsible for the apoptosis process, we chose two: *APAF1* encoding apoptotic protease activating factor-1, a cytoplasmic protein involved in apoptosome formation and activation of caspase 9 in the intrinsic apoptosis pathway [16,17], and *BID* encoding BH3 interacting-domain death agonist, which after activation participates in cytochrome C release from the mitochondrium [18]. Cytochrome C is necessary for apoptosome formation. BID protein is related to the extrinsic apoptotic pathway by the fact that caspase 8 is one of the BID activators. Caspase 8 in turn is a key protease activated in the extrinsic apoptotic pathway (Table 1).

## 2. Results and Discussion

For the purpose of this work, we synthesized selenosteroids **2**–**4** according to a procedure recently presented [8]. The functionalization of commercially available 1,4-androstadiene-3,17-dione (**1**) in a biphasic system was performed in a one-step protocol. The procedure as follows: biphasic system consisting of an equal volume of 10% HCl and ethyl acetate, diphenyl diselenide, and 10 equiv. zinc shaves was stirred until the organic layer was decolorized. Then the liquid phase was transferred under an argon atmosphere to a flask containing 1,4-androstadiene-3,17-dione (**1**). The resulting reaction mixture was stirred at room temperature for 3 h. Synthesis and the structure of seleno-Michael addition products **2**–**4** are shown in Scheme 1.

**Figure 1:**
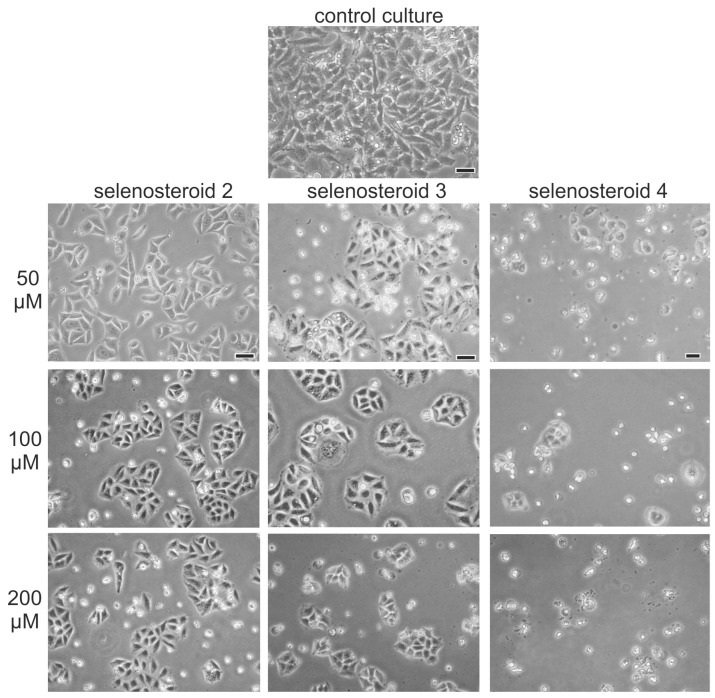
Microscopic observations of HeLa cells after 48 h of culture on a medium with selenosteroids (structure of selenosteroids according to Scheme 1), bar = 20 µm.

First, we explored the antiproliferative activity of SeSt **2**–**4** on the HeLa cancer cell line. Microscopic observation revealed a smaller amount as well as worse condition of cells in cultures with addition of tested SeSt compared with control (Figure 1). After 48 h of experiment on medium with SeSt, we noticed much fewer cells showed adhesion to the surface of the culture vessel and new cell division (compare microscope images, Figure 1). The worst condition of cells was observed on medium with compound **4**, where we found the fewest cells stuck to the culture vessel and the most cells in suspension that did not divide.

**Figure 2:**
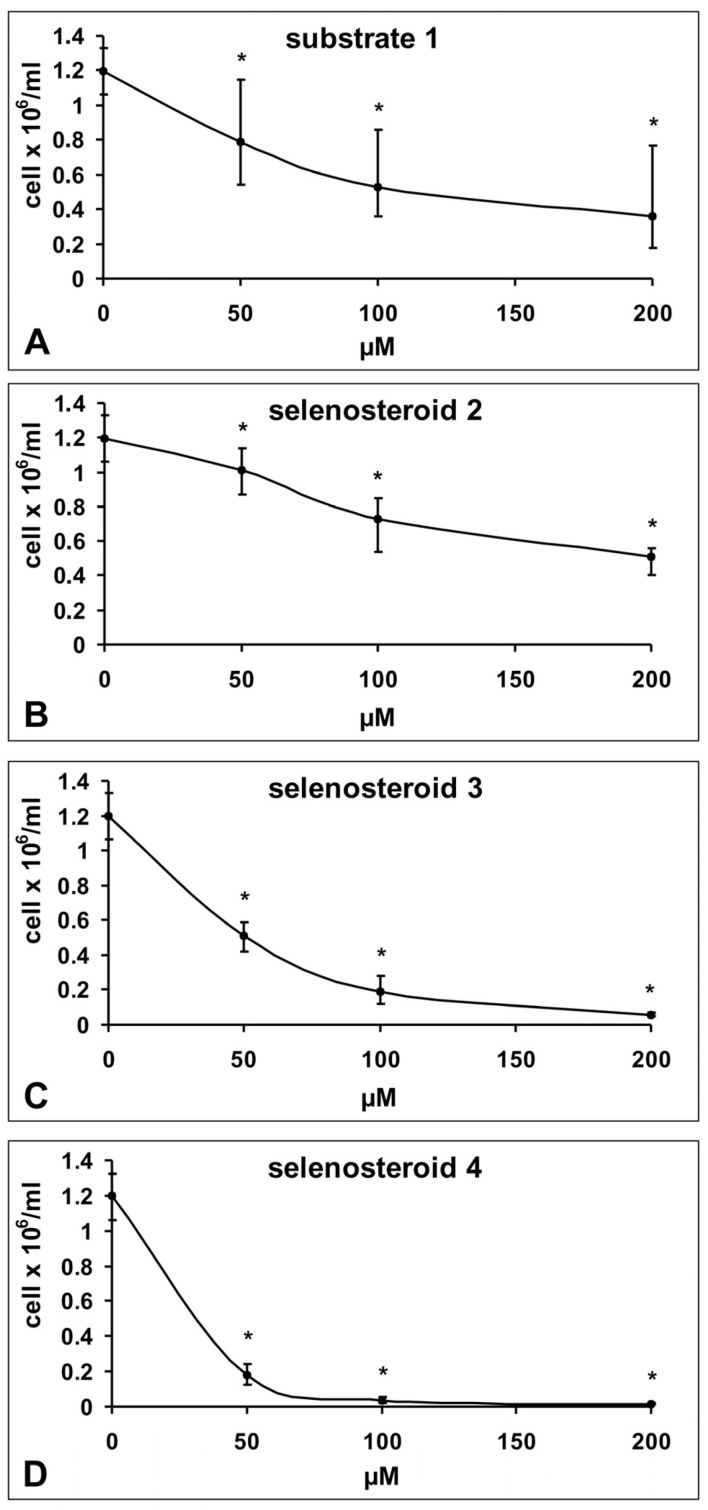
Changes in the number of HeLa cells in culture depending on the concentration of used compound after three days of culture (substrate **1**; 1,4-androstadiene-3,17-dione—substrate for synthesis of selenosteroid **2**, **3**, and **4**, according to Scheme 1). Data represent medians ± maximum and minimum values. Asterisks indicate statistically significant differences compared to the control (Mann–Whitney U-test, *p* < 0.05). The number of cells in the control culture corresponds to zero substrate and selenosteroid concentration.

Analysis of the number of cells after three days of the experiment, when the control culture reached about 90% of confluence, showed significant, concentration-dependent inhibition of the growth rate in a culture with addition of all three tested SeSt (**2**–**4**) and substrate for synthesis (**1**) in the concentration range from 50 to 200 µM. Furthermore, a comparison between the growth rate of HeLa cells in the presence of the substrate (**1**) and the growth rate of cells under SeSt treatment shows that derivatives **3** and **4** exhibit significantly stronger cytostatic effects than substrate (**1**), while the effect of the substrate is comparable to that of SeSt **2** (compare Figure 2A–D). The most effective inhibitor of culture growth in comparison to control was selenosteroid **4** (Figure 2D), where already at a concentration of 50 µM we observed only 15.6% of cells relative to control, and the concentration of 100 and 200 µM almost completely inhibited culture growth. Less effect on cell number was found for selenosteroid **3** (at 50 µM 39.8%, at 100 µM 14.8% of control and at 200 µM almost complete inhibition, Figure 2C). Compound **2** as well as substrate (**1**) had the least influence on culture growth (at 200 µM, 39.0% of control; Figure 2A,B).

**Figure 3:**
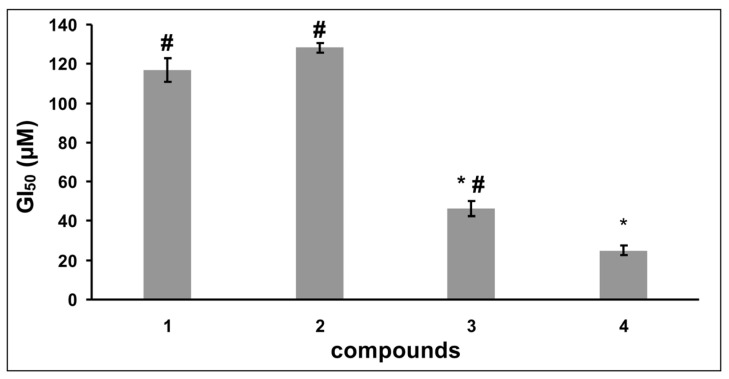
Comparison (means ± SE) of GI_50_ for substrate (**1**) and individual selenosteroids (**2**–**4**) based on data from Figure 2. Asterisks indicate values that differ from substrate **1**, and hashtags indicate values differ from selenosteroid **4** (Anova and RIR post-hoc test *p* < 0.05). The differences in GI_50_ values between selenosteroids **2** and substrate (**1**) were not statistically significant.

Having the data concerning the dependence of the cells growth rate on the concentration of SeSt, we determined the GI_50_ values for each compound (Figure 3). The GI_50_ values were 25.0 µM for **4**, 46.3 µM for **3**, 128.1 µM for **2**, and 117 µM for substrate (**1**). Anova analysis showed that the difference between lowest GI_50_ value for **4** was statistically significant for substrate **1** and both SeSt **2** and **3**. SeSt **4** also acts stronger than **3,** and differences between substrate **1** and SeSt **2** were insignificant (Figure 3). The above results confirm the previous microscopic observations showing that the tested compounds affect HeLa cells with different strength, where the strongest cytostatic effect was that of compound **4**.

**Figure 4:**
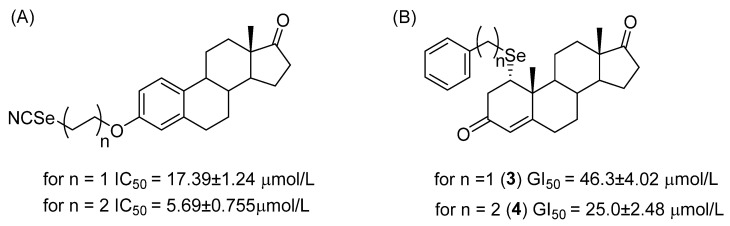
Comparison of IC_50_/GI_50_ values of different selenosteroids taking into account the length of the carbon linker between the selenium atom and the steroid core. (**A**) Structures described in ref. [4]: values given as mean ± SE; lengthening the chain from 2 to 4 carbon atoms resulted in an approximately 3-fold decrease in IC_50_. (**B**) Our experimental data: values given as mean ± SE, where elongation of the carbon chain from 1 to 2 atoms resulted in an approximately 2-fold decrease in GI_50_.

Currently, many selenium derivatives of steroids are known, some of which exhibit cytotoxic properties in relation to cancer cells (IC_50_ from a few to over 100 µM [1,3,4,5,6,10,19]); some of them also exhibit bacteriostatic properties [9]. Concerning HeLa cells, the lowest IC_50_ values are recorded for various A ring-modified selenocyanide and selenourea derivatives [3,4] (IC_50_ from a few to over 50 µM) and a phenylselenourea derivative in the C-3 position of the same ring [1] (IC_50_ approx. 2 µM). Recently, high cytotoxicity of selenocyanate derivatives in which the B ring of the steroid has been modified has been reported (IC_50_ about 6–30 µM) [6]. Among our phenylselenium derivatives at the C-1 position of ring A, the best cytostatic properties show derivative **4** (GI_50_ 25.0 µM) with an extended, two-carbon linker of the phenyl ring with selenium, compared to derivative **3** (almost twice higher GI_50_ in comparison with **4**). It should also be noted that the introduction of chloride into the SeSt significantly deteriorated the cytostatic properties (derivative **2** GI_50_ approx. 130 µM). Comparing literature data concerning the IC_50_ values of various SeSt in relation to breast cancer [2,4,10,19,20] (MCF-7) it can be concluded that these are more resistant to SeSt compared to HeLa cells and that modifications in various positions of the A ring of the steroid are generally most effective, which is also reflected in the results of our study. The exception is the last obtained selenocyanate derivatives at the B ring of the steroid, which in most cases show lower GI_50_ values for MCF-7 in comparison to HeLa [6]. Although the cytostatic properties (GI_50_) of our derivative **4** are slightly weaker than (IC_50_) those of the strongest SeSt known from the literature, it may prove to be prospective in the search for new cytostatic agents. In addition, our results may suggest the role of the length of the carbon chain between selenium and the phenyl ring concerning the cytostatic properties of the compound. Optimization of the length of this linker may be of key importance for the properties of the synthesized derivatives. In the case of our results, extending this linker by one carbon atom reduced the GI_50_ value twofold. The above observation is also reflected in other studies of selenocyanide steroid derivatives (Figure 4) [4,10]. 

The above-mentioned studies showed that the length of the carbon chain between the oxygen at the C-3 and NCSe groups is important for the cytotoxic properties of the derivative. The 2–8 carbon-linker derivatives had better cytotoxic properties compared to the shorter and longer linker derivatives. Therefore, optimization of the length and structure of the linker is a promising direction in the chemical synthesis of derivatives with the best cytostatic properties. If the length of the hydrophobic elements of SeSt derivatives has a significant impact on their cytotoxicity, it can be assumed that the mechanism of SeSt action on cells may be closely related to the interaction with the phospholipid component of cell membranes, leading to disturbances in their structure as cholesterol antimetabolites. This hypothesis is supported by the results of recent studies where it has been shown that some SeSt significantly increase the level of ROS in cells [19].

To propose the mechanism of action of the studied selenosteroids **2**–**4**, we analyzed the profile of cell viability and apoptosis under the influence of these SeSt as well as the expression of genes involved in the mevalonate pathway of steroid synthesis and the process of cell apoptosis.

**Figure 5:**
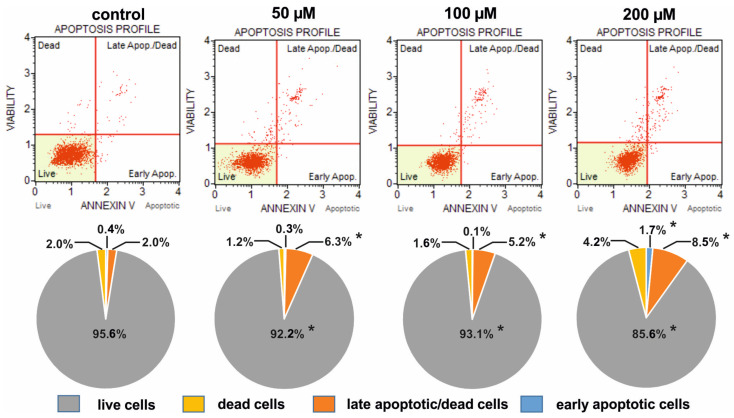
Comparison of the apoptotic profiles of HeLa control cultures and cultures on media with different concentrations of selenosteroid **2** after three days of culture. At the top are examples of histograms of individual cultures obtained as a result of cytometric analysis. At the bottom is the percentage of median data from a series of experiments. Asterisks indicate statistically significant differences concerning the control (Mann–Whitney U-test, *p* < 0.05).

**Figure 6:**
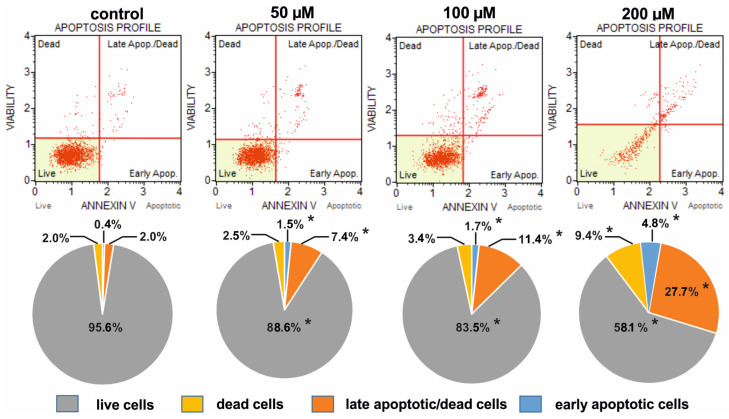
Comparison of the apoptotic profiles of HeLa control cultures and cultures on media with different concentrations of selenosteroid **3** after three days of culture. At the top are examples of histograms of individual cultures obtained as a result of cytometric analysis. At the bottom is the percentage of median data from a series of experiments. Asterisks indicate statistically significant differences concerning control (Mann–Whitney U-test, *p* < 0.05).

**Figure 7:**
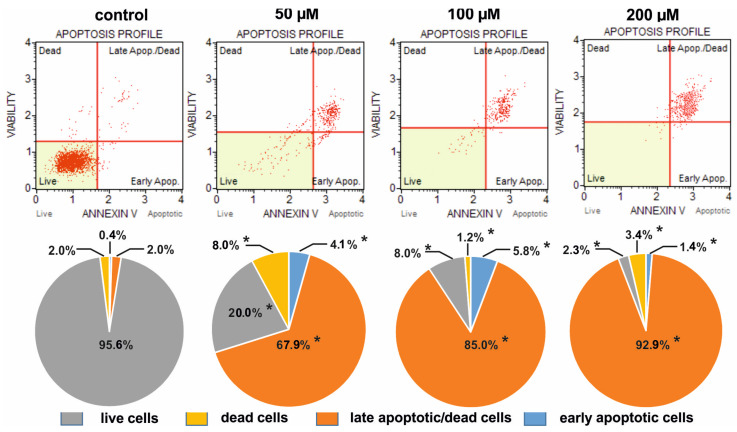
Comparison of the apoptotic profiles of HeLa control cultures and on media with different concentrations of selenosteroid **4** after three days of culture. At the top are examples of histograms of individual cultures obtained as a result of cytometric analysis. At the bottom is the percentage of median data from a series of experiments. Asterisks indicate statistically significant differences concerning control (Mann–Whitney U-test, *p* < 0.05).

Analyzing the survival and apoptosis profiles of cells under the influence of the tested SeSt, we found that all of them have a pro-apoptotic effect on HeLa cells to varying degrees. The observed effect was dependent on the concentration of SeSt (compare Figure 5, Figure 6 and Figure 7).

The weakest pro-apoptotic effect was shown by **2**, where even at a concentration of 200 µM, live cells predominated (85.6%), but still there were fewer of them than in the control (95.6%); at the same time, the number of dead and apoptotic cells increased from 2.0% and 2.4% in control up to 4.2% and 10.2% in culture with **2**, respectively (Figure 5). The strongest pro-apoptotic effect was observed in the case of compound **4** (Figure 7), where at a concentration of 200 µM late apoptotic and dead cells definitely predominated (96.3%), and the number of viable cells decreased to 2.3%. In the case of this compound, a significant increase in apoptotic cell amount together with a strong reduction in the share of live cells was observed already at a concentration of 50 µM (Figure 7). A moderate pro-apoptotic effect was exerted by compound **3**, where substantial changes in the proportion of cells were observed at a concentration of 200 µM. In this case, we found 32.5% apoptotic cells (together early apoptotic and late apoptotic/dead), 9.4% dead cells, and 58.1% viable cells (Figure 6).

There are limited data in the literature on the effect of selenosteroids on the apoptosis process. The pro-apoptotic effect of selenosteroids on HeLa cells in vitro was found in the case of derivatives containing N-phenyl selenourea attached to the C3 position at the steroid molecule [2]. In these studies, approximately 60% apoptotic cells were noted at 25 µM concentration of N-phenyl selenourea steroid derivative, and this result was more than three times higher compared to diosgenin. During the same research, the authors did not find proapoptotic properties of thioureas steroid derivatives. More recently, information on the proapoptotic effect of selenosteroids has also appeared in the work of Huang Y. et al. [5,6,10]. In our studies, we found that selenosteroid **4** at a concentration of 50 µM caused apoptosis of about 72% of cells. In culture while compound **2** (organohalogen moiety linked to selenium) has a much lower proapoptotic effect. The intensity of the effect we observed was also correlated with the length of the carbon linker between the selenium attached to the C1 carbon of the A ring of the base steroid. A much stronger pro-apoptotic effect was noted in the case of derivative **4**, which has a longer linker compared to derivative **3**. The pro-apoptotic effect was also tested on steroidal ethynyl selenide derivatives where the selenium-containing residue was combined with the C-12 of the C ring, the C-6 of the B ring, or the C-17 of the D ring [17]. The last derivative has the highest proapoptotic effect (almost three-fold increase in the activity of caspases 3/7 in MCF-7 cells at a concentration of about 200 µM compared to the control). Due to the different methodology and structure of the studied SeSt, it is not easy to compare our results with the data from the literature; however, it can be concluded that the derivative **4** studied in this work has similar pro-apoptotic properties to other SeSt known from the literature. In addition, our results regarding the reduction in cell growth rate and promotion of the apoptosis process indicate that a good direction of chemical syntheses to improve the cytostatic properties of the tested SeSt will be the optimization of the structure and length of the carbon link between the selenium atom and phenol moiety in the described group of SeSt derivatives.

**Figure 8:**
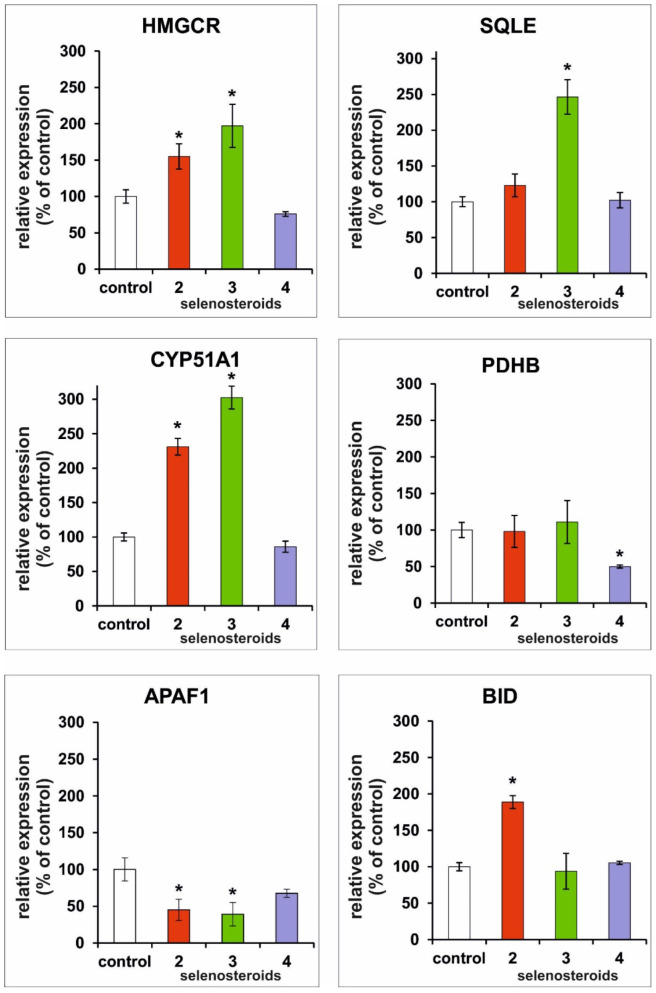
Comparison of the relative expression of selected genes after three days of HeLa cell culture in media supplemented with different selenosteroids (**2**—red color, **3**—green color, **4**—purple color) at a concentration equal to the GI50 value as shown in Figure 3. Values are shown as % of control ± standard deviation. Asterisks indicate statistically significant differences compared to the control (Student’s *t*-test, *p* < 0.05).

The in vitro cultures of HeLa cells with three tested SeSts enabled us to compare the relative expression profiles of genes (Figure 8) responsible for apoptosis induction with those related to cholesterol synthesis. Of the SeSt tested, the strongest effect for increase of cholesterol synthesis-involved genes expression (*HMGCR*, *SQLE*, *CYP51A1*) was observed for compounds **2** and **3** compared to cells cultured in the presence of compound **4** and the control culture. In contrast, *PDHB* gene expression was significantly decreased in culture with **4**, while the other compounds did not change their expression relative to the control. The presence of compound **2** in the culture medium increased the expression of the pro-apoptotic gene *BID* in HeLa cells compared to cells cultured in the presence of compounds **3** and **4** where the level of this transcript did not significantly change compared to the control. However, in the case of the *APAF1* gene, whose product is an essential factor of the apoptosome [16], we found a reduction in its expression under the influence of compounds **2** and **3**, as well as a tendency for reduction of its expression in the case of compound **4** in comparison with the control (Figure 8).

The results of transcripts-level analysis of selected genes of the cholesterol synthesis pathway and those involved in the apoptosis process allowed us to make some observations that may contribute to verifying our hypothesis. Results obtained after three days of HeLa cell culture may suggest different mechanisms of action of tested SeSt. Compounds **2** and **3**, which have a milder effect on HeLa culture, act in the same way, while the most potent compound, **4**, may also have additional mechanisms of action.

The significant increase in *HMGCR*, *SQLE*, and *CYP51A1* gene expression in cultures with compounds **2** and **3** indicates the influence of SeSt on the synthetic rate of the mevalonate pathway in HeLa cells. Increased transcription levels of these key enzymes in the pathway indirectly support our hypothesis about the incorporation of these SeSt into the cell membrane and its destabilization. The key role of mevalonate pathway in tumor development is well known [13,21]. Although the activating effect of these compounds on cholesterol synthesis seems to be evident, the observed increase in expression of mevalonate pathway genes may be a specific response of the cell to membrane destabilization (increase in the rate of endogenous cholesterol synthesis should compensate for the membrane’s destabilization). Overexpression of mevalonate pathway-involved genes (especially *SQLE*) was reported in different types of cancer cells [22,23,24,25,26].

The membrane destabilization may cause activation of the extrinsic apoptosis pathway, which may be indicated by overexpression of the *BID* gene. The BID protein is related to that pathway because it is activated by caspase 8, which is the key protease for the extrinsic apoptosis pathway [12]. Procaspase 8 may be part of two complexes related to the induction of apoptosis. The initial plasma membrane-bound complex (complex I) and cytoplasmic complex (complex II) are known as death-inducing signaling complexes [27]. Complex I is directly related to the induction of the extrinsic apoptotic pathway, while complex II triggers an amplified cascade of caspase activation via crosstalk with the intrinsic apoptosis pathway [28], probably by the release of proapoptotic factors from mitochondria. The observed reduction in *APAF1* expression, which is crucial for triggering the intrinsic apoptosis pathway (activation of caspase 9), may be a defensive reaction of cancer cells [29].

Taking into consideration the above-proposed mechanism, it can be assumed that the cytotoxic effect of the studied SeSt results from their interaction with the cell membrane and induction of the extrinsic apoptosis pathway.

SeSt **4** in the tested experimental system (concentration equal to GI_50_) does not affect the expression of genes directly related to the mevalonate pathway as well as the proapoptotic *APAF1* and *BID* genes. Taking into account the strongest cytostatic effect of this SeSt, it can be concluded that in this case, the cells will not have time to activate defense mechanisms, as in the case of less efficient derivatives **2** and **3**. It is noteworthy that *PDHB* expression is reduced under the influence of SeSt **4**. The protein encoded by *PDHB* is a key component of the pyruvate dehydrogenase complex [30] that catalyzes the oxidative decarboxylation of pyruvate, which is one of the main sources of acetyl-CoA used in both catabolic (Krebs cycle) and anabolic reactions (e.g., steroid synthesis). Inhibition of the above-mentioned reaction leads to a decrease in the level of ATP and a reduction in lipid synthesis, which further also contributes to the disorganization of the cell membrane. The limitation of this reaction may be the main reason for the strongest anti-proliferative effect of derivative **4**. Comparing the data in Figure 8 and apoptotic profiles (Figure 5, Figure 6 and Figure 7), it can be assumed that derivative **4**, in addition to affecting the membranes, acts inside the cell in a way similar to steroid hormones, limiting the expression of PDHB, which may additionally enhance the proapoptotic effect of this derivative.

**Figure 9:**
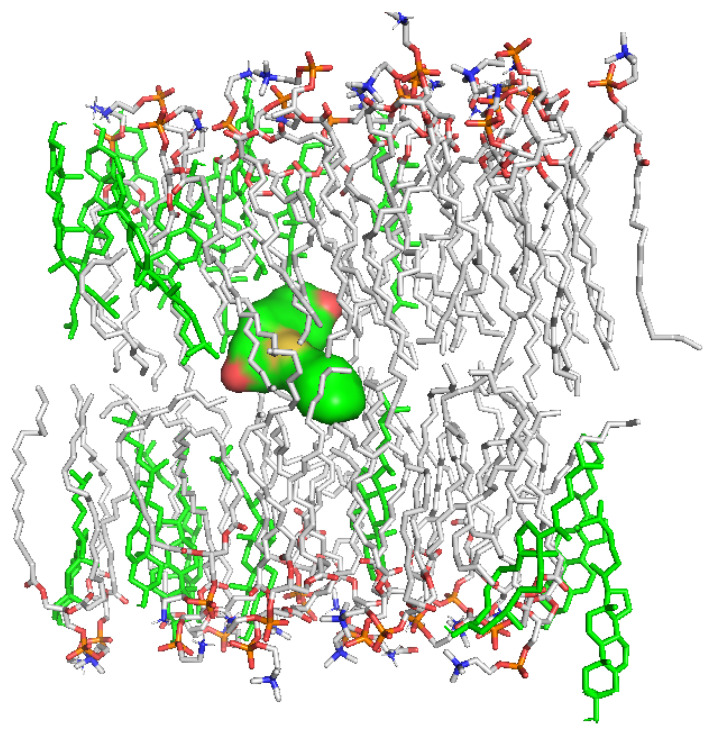
Position of selenosteroid **4** (surface view) in the lipid bilayer model of HeLa lineage cells. Cholesterol molecules forming the layers are highlighted in green. The water molecules and ions were removed for the sake of clarity.

To assess the effect of obtained SeSt on the stability of the membrane bilayer of the HeLa cells, a series of in silico experiments were performed, starting with molecular docking. As detailed below in the “Experimental” section, the procedure involved repeated dockings with the box shifting to capture all the diversity in the membrane structure. For every box position, docking calculations were performed to generate the best-scored (i.e., with the lowest value of Δ*G*) poses within. A number of poses, substantially differing in placement, were obtained as a result. The best docking scores for SeSt **2**, **3**, **4** are −10.4, −9.6, and −11.6 kcal/mol, respectively. This suggests the most efficient membrane penetration by SeSt **4**, which is in agreement with the in vitro experiments discussed above. Because of the best affinity, a visualization of the optimal pose for **4** is shown in Figure 9 below (in order to improve clarity, Figure 9 does not contain the SeSt **2** and **3**). It is seen that SeSt **4** is deeply buried in the membrane structure, filling the empty space between the layers. Similarly, the best poses for SeSt **2** and **3** are also located here. A significant variation (of about 5–6 kcal/mol) in docking scores between these favorable (between the layers) and other (inside the layers) positions is noted. The behavior of cholesterol is different. Sites comparable to the favorable ones for the new derivatives (i.e., those located between layers) are scored by poor ratings of the order of −(5–7) kcal/mol. The best affinities (~−9 kcal/mol) are noted at the membrane edges. These results may suggest a different mechanism for the penetration of the membrane by cholesterol and proposed chemicals. Indeed, while cholesterol is embedded inside the phospholipid layers, the new compounds are rather deposited between these layers. The consequence may be the improper functioning of the penetrated membrane leading to its destabilization and, ultimately, cell apoptosis. Because of its best docking score, the most effective agent promoting this process appears to be SeSt **4**.

**Figure 10:**
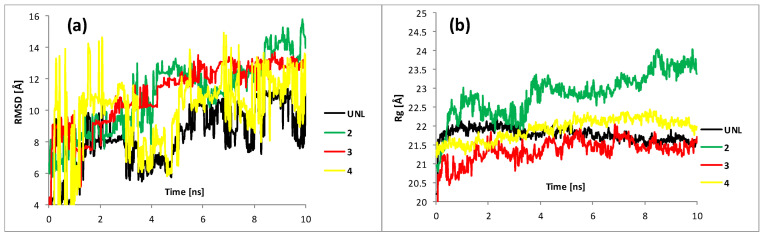
Analysis of RMSD and Rg of unliganded HeLa bilayer model (UNL) and three complexes during 10 ns of molecular dynamics simulation: (**a**) root mean square deviation (RMSD) for non-hydrogen atoms; (**b**) radius of gyration (Rg).

In order to further understand the mechanism of interaction of the proposed derivatives with the HeLa cell membrane, 10 ns molecular dynamics simulations were carried out for both the membrane alone and the complexes with SeSt **2**–**4** embedded. The starting point, resulting from docking, was located between two monolayers of the membrane (see Figure 9 above). Visual inspection of the resulting trajectory indicates a movement of the ligand between the membrane-forming layers rather than their penetration. Due to the significant size of the ligand molecule, this is an expected result. In order to quantify the impact of the observed motion on the stability of the membrane, the change in RMSD (root mean square deviation) and Rg (radius of gyration) values along the trajectories were analyzed; results are plotted in Figure 10. RMSD analysis permits for assessing the system’s stability during simulation. The averaged RMSD values of 8.2, 11., 11.1, and 10.2 Å for UNL and SeSt **2**, **3**, and **4**, respectively, are much higher than their counterparts for proteins, with typical average RMSDs of 2–5 Å [31,32]. This is not surprising since the cell membrane, consisting of noncovalently bonded phospholipids and sterols, is a much more labile structure than proteins. The interrelationships of the RMSDs for the different systems are important for assessing stability. From a comparison of the average values and from Figure 10a, it can be seen that ligand embedment in the membrane causes an observable increase in RMSD. The magnitude of this effect changes with time, although after 5 ns it appears to stabilize at about 2 Å. Although the influence of SeSt **4** appears to be smaller than that of the other two, there is a noticeable scattering of the interaction strength in the graph. In fact, the values of standard deviation (SD) and coefficient of variation (*h* = SD/mean) are by far the largest for SeSt **4** and the smallest for the ligand-free form. This may indicate that the actual influence of this compound on membrane stability is more significant than suggested by the RMSD analysis. On the other hand, the influence of all tested SeSt on membrane destabilization is apparent. The radius of gyration (Rg), defined as the root mean distance from the axis of gyration to a point where the total mass of the structure is concentrated, is considered a gauge of the compactness of the system. Its increase may indicate a destabilization of the whole structure—for example, the unfolding of the protein or disintegration of the lipid bilayer. The Rg for the structures considered here is plotted in Figure 10b. Its magnitude is initially utmost for the membrane without ligands. Over time, the relationship changes, and by the end of the simulation the unliganded form shows the smallest Rg. Except for SeSt **2**, the differences are not significant; however, Rg of SeSt **2** is surprisingly large and increases with time, which may indicate faster disintegration of the membrane. It seems to be consistent with the time evolution of RMSD, which reaches its highest value after the ninth second. A thorough clarification of this probably requires longer simulations to be carried out, however.

## 3. Conclusions

**Figure 11:**
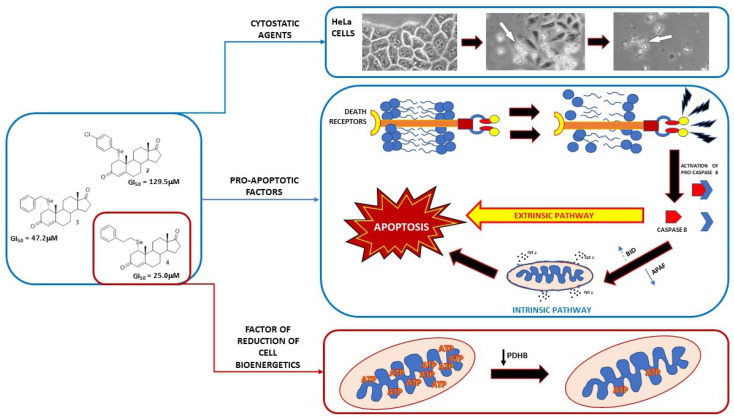
Proposed mechanism of action of the tested selenosteroids **2**–**4**. All tested compounds exhibit cytostatic activity with varying intensity depending on their chemical structure. The proapoptotic effect of selenosteroids may result from the destabilization of the cell membrane and activation of the extrinsic apoptotic pathway related to caspase 8. Elevated expression of BID activated by caspase 8 may enhance apoptosis by releasing cytochrome C from mitochondria. The most cytostatic derivative, **4**, additionally reduces PDHB expression, probably through a hormonal pathway, which leads to the disorganization of cell bioenergetics and inhibition of the mevalonate pathway by reducing acetyl-CoA synthesis.

Our SeSt **4** has similar properties to other known selenosteroids and therefore can be considered as a prospective cytostatic agent. The results of our research, in contrast with the literature, allow us to conclude that the incorporation of selenium substituents into the A ring of steroid can give better cytostatic results against cancer cells, and the length of the carbon linker in the case of phenylselenium or selenocyanate derivatives is important for their action. The tested derivatives probably interact with the cell membrane as cholesterol antimetabolites, causing its disorganization and activation of the extrinsic apoptosis pathway, which may be evidenced by an increase in *BID* expression and reduction in *APAF1* expression (Figure 11). Moreover, our hypothesis has been confirmed by in silico experiments. Molecular docking proves that all SeSt penetrate the cell membrane by easily embedding between its layers. Among tested SeSt, the most favorable Δ*G* value is shown by **4**, which suggests its most efficient penetration of the bilayer structure. Molecular dynamics studies indicate a significant membrane destabilization (which may lead to cell apoptosis) after embedding of each of the compounds **2**–**4**. Derivative **4** penetrates the membrane most readily; for that reason, it appears to be the most potent in membrane disorganization of HeLa cells. In the case of the most potent derivative, **4**, an additional, intracellular mechanism is possible, causing a reduction in cell bioenergetics due to a decrease in *PDHB* expression.

## 4. Experimental Section

### 4.1. Synthesis

Compounds **2**–**4** were obtained according to the described protocol [11] as follows: Diselenide (0.13 mmol) was added to a flask with 2 mL of 10% HCl, 2 mL of ethyl acetate, and 10 equiv. of zinc shaves. The reaction was stirred vigorously until the discoloration of the organic layer occurred. Then the liquid was transferred into another flask under inert conditions (Ar) and the steroid (0.2 mmol) was added. The reaction mixture was stirred for 3 h. Then the reaction mixture was poured into water and extracted with ethyl acetate (3 × 20 mL). The organic layer was dried with Na_2_SO_4_, filtered, and the solvent removed under vacuum. The products were purified by flash chromatography. Obtained 1α-(4-chlorophenylselenyl)-androst-4-en-3,17-dione (**2**), 1α-benzylselenylandrost-4-en-3,17-dione (**3**), and 1α-phenylethylandrost-4-en-3,17-dione (**4**) were proved identical in all respects with the same compounds described in the literature [11].

### 4.2. Cells Culture

For evaluation of the impact of three tested SeSt (compound **2**, **3**, and **4**; Scheme 1) on cell growth and apoptosis, we used an in vitro culture of HeLa (ATCC-CCL-2) cells. Cultures were maintained in a NuAire NU-5820E incubator (37 °C, concentration of CO2: 5%, humidity: 95%) on medium MEM199 (Gibco ref. no. 31150-022), with 10% Fetal Bovine Serum and antibiotics (penicillin 50 U/mL, streptomycin 0,05 mg/mL). The initial culture density was approximately 1 × 10^5^ cells/mL. Initially, all cultures were maintained for 24 h on control medium without SeSt. Subsequently, in control cultures the medium was replaced with fresh, whereas in experimental variants the medium was replaced with SeSt-containing in concentrations 50, 100, and 200 µM (first we prepared stock solutions with DMSO and added to the medium in volume not exceeding 100 µL). Final concentration of DMSO in the medium did not exceed 0.2%. According to our knowledge, this DMSO concentration is neutral to cells in vitro [33,34,35]). All cultures were grown in 6-well plates (one plate for each combination) until the control variant reached 90% of confluence (about 3 days). The cells were then harvested using trypsin-EDTA solution (Sigma ref. no. T3924) and suspended in the same volume of phosphate buffered saline (Sigma ref. no. D8537). For cell counting, we used automatic cell counter EVE-MT (NanoEnTec Inc., Seoul, Korea). Quantity assessment of live/dead with division of early and late apoptotic cells was performed with Merck Millipore Muse™ Cell Analyzer (0500-3115) using Muse™ Annexin V & Dead Cell assay kit (Cat. No. MCH100105), according to the manufacturer’s instructions. During the experiment, observations of cultures were carried out using the Olympus inverted contrast phase microscope (CKX 41).

For the study of the relative expression of selected genes (see below), HeLa cultures were maintained as above, on media with SeSt concentrations equal to their GI_50_ values determined based on the cell growth curves plotted for individual SeSt.

### 4.3. Relative Gene Expression Analysis

Total RNA was isolated and purified using an RNeasy Mini Kit (Qiagen, Hilden, Germany) following the manufacturer’s protocol. Total RNA integrity was verified by 1.5% agarose gel electrophoresis, identified by ethidium bromide staining and OD_260/280_ absorbance ratio > 1.95 using a Nano-Drop ND-2000 spectrophotometer (ThermoFisher Scientific, Waltham, MA, USA). cDNA synthesis was performed using a High Capacity cDNA Reverse Transcription Kit (ThermoFisher Scientific, USA) on 1 µg of total RNA in the T-100 Thermal Cycler (Bio-Rad, Hercules, CA, USA).

The relative expression analysis of studied genes were measured by quantitative real-time PCR using commercially designed human QuantiTect Primer Assays (Qiagen): Hs_CYP51A1_1_SG (cytochrome P450 family 51 subfamily A member 1), Hs_SQLE_1_SG (squalene epoxidase), Hs_HMGCR_1_SG (3-hydroxy-3-methylglutaryl-CoA reductase), Hs_APAF1_1_SG (apoptotic peptidase activating factor 1), Hs_BID_1_SG (BH3 interacting domain death agonist), Hs_PDHB_1_SG (pyruvate dehydrogenase E1 subunit beta), and Hs_PRS18_1_SG (s18) as normalizer and an endogenous control. Real-time PCR reaction was performed in duplicate using the SYBR Green PCR Master Mix (Qiagen, Germany) carried out in the CFX OPUS Real-Time PCR System (Bio-Rad, Hercules, CA, USA). The thermal cycling conditions followed the manufacturer’s instructions. At the end of the amplification phase, a melting curve analysis was carried out on the products formed to check their specificity. Based on the standard curve (which was generated employing a serial of five dilutions of cDNA derived from unstimulated cells in reaction with the house-keeping gene—s18), the levels of studied genes transcripts were calculated after normalization of amplification products to s18. We used the comparative C_T_ method for relative quantification (ΔC_T_ method) to calculate our data.

### 4.4. Statistical Analysis

Data represent the number of cells from 8–14 independent experiments. Data representing apoptotic profile and relative gene expression came from at last 4 independent experiments. The results were evaluated using the Shapiro–Wilk W-test to identify normal distribution of data and the Levene L-test for testing of homoscedastic variances. In the case of normal distribution of data and homoscedastic variances, t-Student test or Anova with RIR post-hoc test were used to compare the mean values. In the case of non-normal distribution of data, we used the nonparametric Mann–Whitney U-test to compare median values.

### 4.5. Molecular Docking

**Figure 12:**
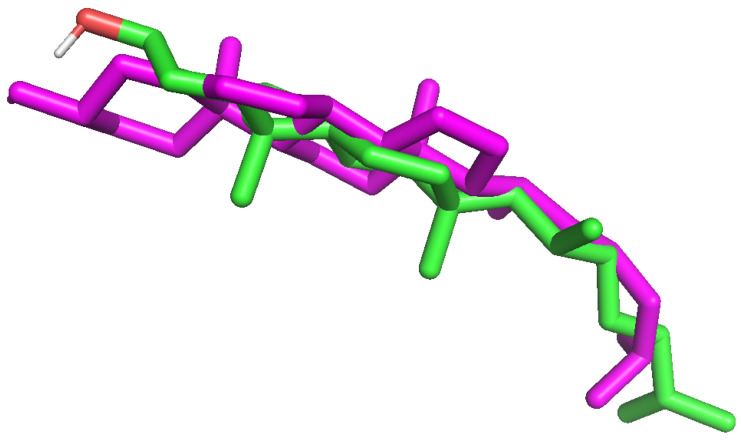
The alignment of the built-in cholesterol moiety from ref. [43] (green scaffold) superimposed on the best pose from the redocking procedure (magenta).

Molecular docking studies were conducted to better understand the mechanism of interaction of the proposed derivatives with the cell membrane using the latest (1.2.5) version of the AutoDock Vina program [36]. The Pymol [37] molecular visualization package was utilized for the presentation of the results. The Membrane Builder [38] application from the CHARMM-GUI [39] online environment was used to create, solvate, and ionize the bilayer mimicking the membrane of the HeLa cell. This buildup was based on data from ref. [40], proposing the HeLa mimicking model (POPC:POPE:POPS:CHOL = 29:31:6:34, where POPC stands for phosphatidylcholine, POPE—phosphatidylethanolamine, POPS—phosphatidylserine, and CHOL—cholesterol). The prepared model was pretreated for docking by cleansing its structures from solvent and ions, adding polar H atoms and Kollman charges [41]. The cubic box of 20 × 20 × 20 Å is set up to encapsulate the ligands during the docking procedure. The problem is to locate the optimal site to insert the ligand into the membrane. Typically, reference compounds are embedded in crystallographic structures; their location points out the active center. However, this is not the case here. Because of that, docking calculations were performed on a series of overlapping grid boxes covering the entire area of variation of the bilayer features. Since the lipid chains are oriented along the *z*-axis, the box moved both along this axis and in the xy-plane. The best-score poses were automatically detected. This was done with an in-house submitting script, repeating the calculations over the subsequent grid boxes, available from the authors upon request. Since there are no reliable crystallographic structures, validation of the docking protocol is problematic and must be approximated. To carry it out, we chose a membrane model similar to the HeLa mimicking models (POPE + POPC with 30 molar-% cholesterol) resulting from exhaustive (300 ns) molecular dynamics simulations [42,43] and validated against reliable experimental data. For that reason, it is believed that the arrangement of cholesterol moieties properly reflects the real structure. Validation involved removing one cholesterol molecule from the membrane and docking it again (redocking). A satisfactory agreement of the resulting conformation with the original one was found with RMSD = 2.045 Å (see Figure 12 below); thus, the identical methodology was subsequently used to investigate the new derivatives.

### 4.6. Molecular Dynamics

Molecular dynamics modeling was carried out using the NAMD 2.14 and VMD 1.9.4 programs [44,45]. The simulations were started with the best poses generated in the docking experiments. Every run began with an initial minimization for 50,000 steps, followed by a gradual heating from 0 to 310 K in 2 K steps and an additional 20,000 steps of equilibration. Preequilibrated structures were then subjected to a 10 ns production run with a timestep of 2 fs, and frames were written to the .dcd file for every 10,000 steps. Scripts provided by the NAMD/VMD developer were utilized to obtain the RMSD/Rg values for resulting trajectories. The Langevin piston methodology enforced a constant pressure of 1 atm with a decay period of 100 fs; a temperature of 310K was imposed by Langevin dynamics with a damping factor equal to 5 (1/s).

## Figures and Tables

**Table 1:** Characteristics of the genes for which expression was studied (based on www.ncbi.nlm.nih.gov (accessed on 28 October 2022); www.genecards.org (accessed on 30 October 2022)).

Types of Genes	Gene/Locus	Function of Encoded Protein
Related to cholesterol synthesis	*HMGCR*/5q13.3	3-hydroxy-3-methylglutaryl-CoA reductase—a transmembrane enzyme, bound to endoplasmic reticulum; catalyzes the conversion of (3*S*)-hydroxy-3-methylglutaryl-CoA to mevalonate, which is the rate-limiting step in the synthesis of cholesterol and other isoprenoids, playing a significant role in cellular cholesterol homeostasis; is regulated by a negative feedback mechanism through sterols and non-sterol metabolites derived from mevalonate.
	*SQLE*/8q24.13	squalene epoxidase—an enzyme with monooxygenase activity; catalyzes the stereospecific oxidation of squalene to (*S*)-2,3-epoxysqualene—the first oxygenation step in sterol biosynthesis.
	*CYP51A1*/7q21.2	lanosterol 14-alpha-demethylase, monooxygenase— a member of the cytochrome P450 superfamily (Cytochrome P450 Family 51, Subfamily A, Member 1); is an endoplasmic reticulum protein which catalyzes reactions involved in the synthesis of cholesterol, steroids, and other lipids; is an essential housekeeping enzyme and is evolutionarily highly conserved.
	*PDHB*/3p14.3	pyruvate dehydrogenase E1 subunit beta—is a nuclear-encoded mitochondrial multienzyme complex member that catalyzes the overall conversion of pyruvate to acetyl-CoA and carbon dioxide; provides a link between glycolysis and the tricarboxylic acid cycle.
Related to apoptosis	*APAF1*/12q23.1	apoptotic peptidase activating factor 1, a cytoplasmic protein that initiates apoptosis through the autocatalytic activation of procaspase 9 (dependent on ATP) and then caspase 3; is a component of the apoptosome
	*BID*/22q11.21	BH3 interacting domain death agonist, a proapoptotic protein that regulates apoptosis through binding to proteins BAX and /or BCL2; protein is activated by caspase-8, which is activated in the extrinsic apoptosis pathway; the BID protein is associated with cytochrome c release from mitochondria, thereby amplifying the apoptosis signal and contributing to the activation of the apoptosome.

## Data Availability

Data will be available on request.
